# Supplementary figures and images for: Patient-reported outcomes predict return to work and health-related quality of life six months after cardiac rehabilitation: Results from a German multi-centre registry (OutCaRe)
Source: PLoS One. 2020 May 5;15(5):e0232752. doi: 10.1371/journal.pone.0232752 (PMC7199966; doi:10.1371/journal.pone.0232752)

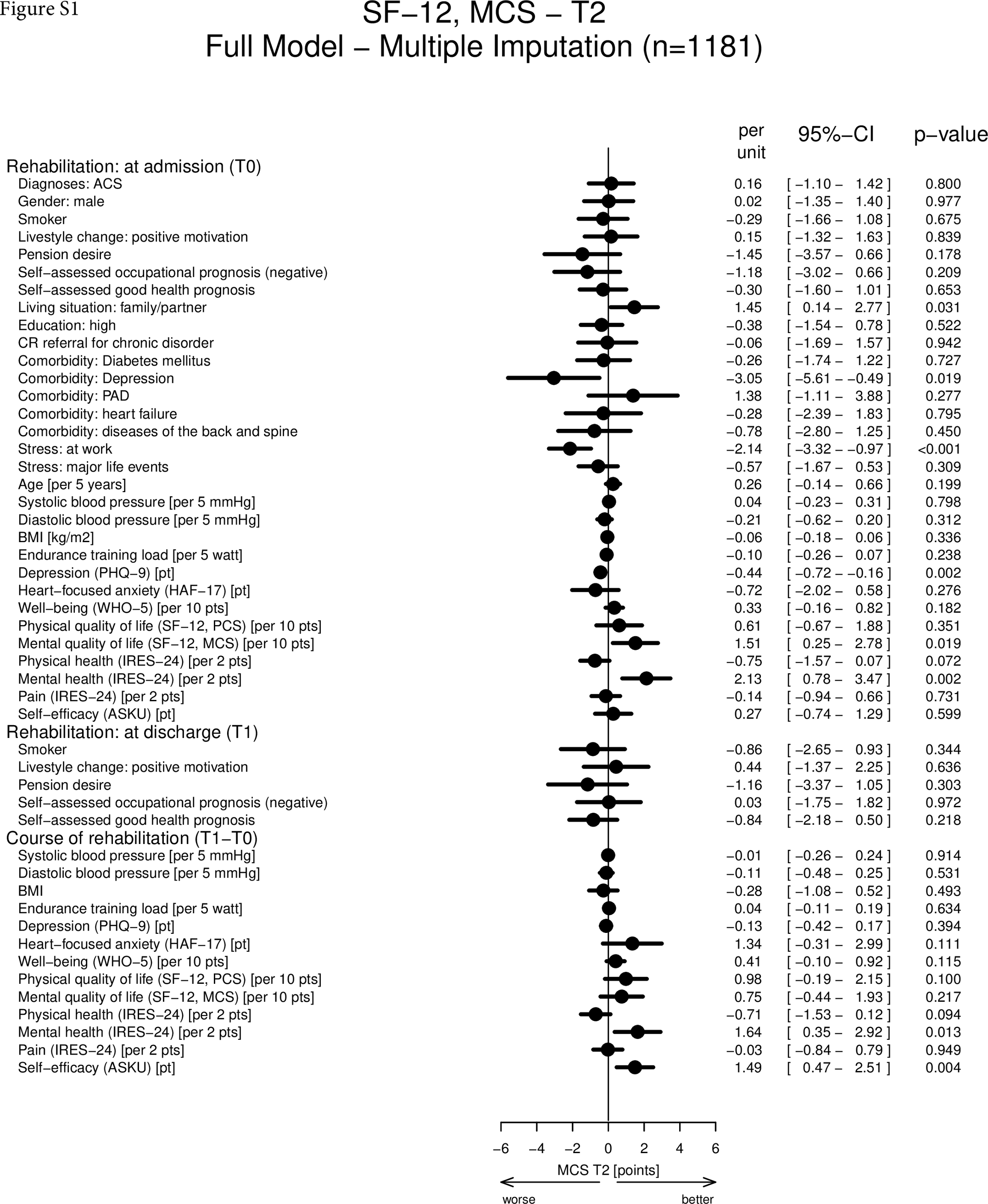

Supplement: S1 Fig — SF—12, MCS—T2. (TIF) [file pone.0232752.s001.tif]

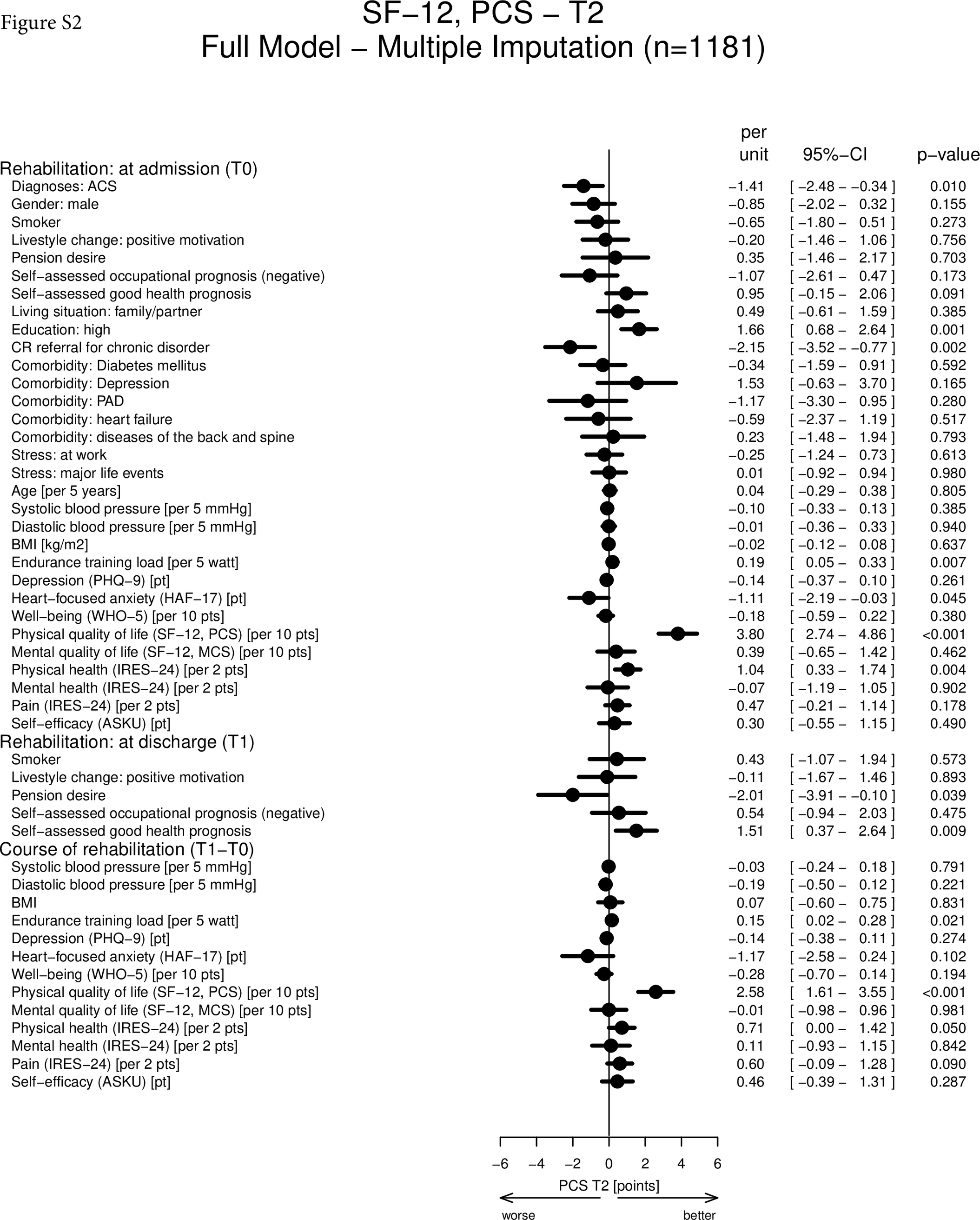

Supplement: S2 Fig — SF—12, PCS—T2. (TIF) [file pone.0232752.s002.tif]

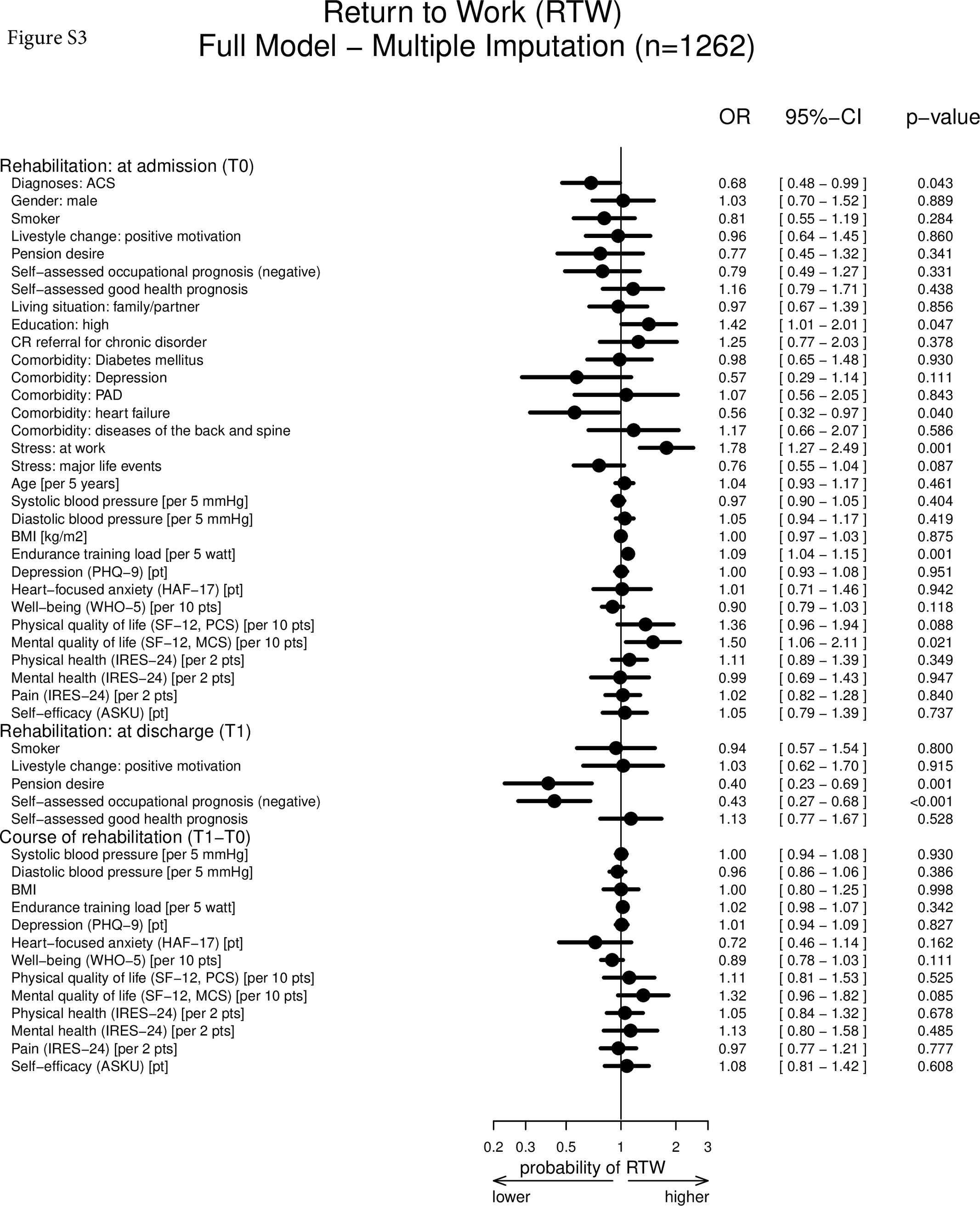

Supplement: S3 Fig — Return to work(RTW). (TIF) [file pone.0232752.s003.tif]

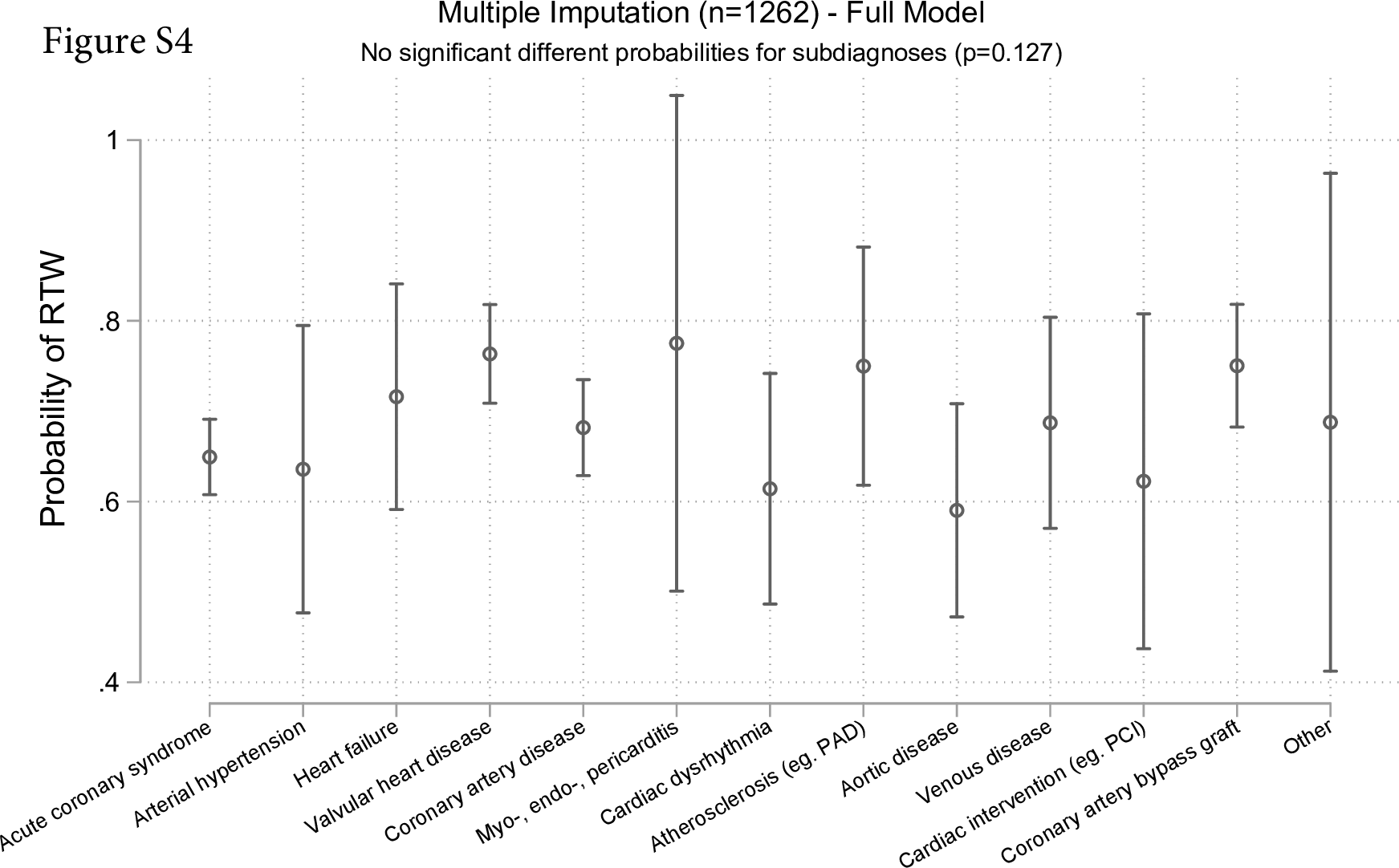

Supplement: S4 Fig — No significant different probabilities for subdiagnoses (p = 0.127). (TIF) [file pone.0232752.s004.tif]
