# Supplementary material for: Patient-reported outcomes predict return to work and health-related quality of life six months after cardiac rehabilitation: Results from a German multi-centre registry (OutCaRe)
Source: PLoS One. 2020 May 5;15(5):e0232752. doi: 10.1371/journal.pone.0232752 (PMC7199966; doi:10.1371/journal.pone.0232752)
Supplement: Attachment — Submitted filename: Review_R1.docx [file pone.0232752.s006.docx]

I would like to compliment the authors on their revisions. The document has greatly improved. Nevertheless, I still have some remarks that I hope the authors are willing to take into consideration. I mainly feel that the discussion section could be further improved.

**Major remarks**

1. The introduction greatly improved as compared to the previous version. Nevertheless, I am still not convinced of the added value of investigating predictors of HRQL. The introduction states that there are mainly inconclusive results with regard to return to work with suboptimal outcomes after CR. Lines 85-88 seem to suggest that predictors for HRQL are already known. In line 73 the authors state that return to work influences HRQL, but why do the authors decide to separately investigate predictors of return to work AND HRQL and not just return to work?
2. A clear research aim/goal is missing in the introduction
3. In the statistics sessions the MID is mentioned for part of the patient-reported outcomes, but not for all. How did you deal with the other outcomes, such as the WHO-5, ASKU and Wurzburger list?
4. The discussion sessions has greatly improved with your extra information, but is now quit long. I suggest to shorten this section and also rearrange a bit the paragraphs. Start with discussing the predictors that you found and how you think (based on literature) these predictors could influence RTW/QOL (this last information is for instance missing for heart-focused anxiety). Move the paragraph lines 349-358 to the end and combine this paragraph with lines 382-389. Describe in this paragraph not only what is NOT working for RTW & HRQL, but also give clear suggestions (based on your outcomes) what should be done to improve CR.
5. There seems to be some grammar errors in the paper. I recommend consulting a native speaker/ professional to check the paper.

**Smaller remarks**

1. In the abstract ‘lifestyle change motivation’ is defined as risk factor management, shouldn’t this be part of patient-reported outcomes?
2. I advise to split lines 42-46 (abstract) in two sentences to increase readability
3. Please make clear in your abstract what predictors were measured at discharge and which predictor concern changes during CR.
4. Could you add a reference to line 73 that return to work influences HRQL?
5. Line 74-77 it is unclear whether outcomes or predictors are discussed (e.g. functional status is a predictors and HRQL an outcome?). I advise to delete this sentence.
6. Please also add to your methods section that you check for multicollinearity.
7. Sex and gender are both used in your document. Please pick one (I prefer sex).
8. I would recommend adding a subheading “Predictors” to page 6 to increase readability.
9. Nice summary at the start of the discussion. Could you shortly add (one sentence) how RTW and QOL were improved at 6 months follow-up?
