# Supplementary material for: Patient-reported outcomes predict return to work and health-related quality of life six months after cardiac rehabilitation: Results from a German multi-centre registry (OutCaRe)
Source: PLoS One. 2020 May 5;15(5):e0232752. doi: 10.1371/journal.pone.0232752 (PMC7199966; doi:10.1371/journal.pone.0232752)
Supplement: Attachment — Submitted filename: 2020-03-16_OutCaRe_RTW-QoL_ReviewerResponseII.docx [file pone.0232752.s007.docx]

**PONE-D-19-29345R1**

**Patient-reported outcomes predict return to work and health-related quality of life 6 months after cardiac rehabilitation: Results from a German multi-centre registry (OutCaRe)**

**Responses to the Editor and Reviewers’ Comments**

*We thank the editor and the reviewers for re-reviewing our manuscript. Please find our responses to the comments below. Changes in the manuscript text are highlighted in grey.*

*Sincerely,*

*Annett Salzwedel
for the authors of the manuscript.*

Editor:

-See my previous comment #10 concerning the RTW analysis: I am still not completely clear why those who were not working at baseline were not excluded.

*A large percentage of those undergoing rehabilitation generally work, but have been on sick leave since their cardiac event and the corresponding hospitalization until rehabilitation and beyond. Cardiac rehabilitation in Germany has the mission per law to support RTW for patients in employable age even if they were at sick leave or unemployed before rehab. Our choosen method represents this real world goal of rehabilitation in Germany. We tried to clarify this fact in the methods:* Return-to-work was analyzed regardless of the employment status at baseline as it is a goal of CR in Germany to support return-to-work even in patients at sick leave or unemployed before CR. (line 201-203)

-Previous comment #15 concerning figures 2 and 3: in their description please list all variables in the models.

*As requested, we now listed all variables in the description of the figures as follows:*

The forest plot shows the final model after backward selection. The following variables were taken into account in the starting model (see supplemental material, figure S1-S3): at admission to rehabilitation: sex, smoking, lifestyle change motivation, pension desire, self-assessed occupational prognosis, self-assessed health prognosis, living situation, educational level, rehabilitation referral for chronic disorder, comorbidities (diabetes mellitus, depression, peripheral artery disease, heart failure, diseases of the back and spine, stress at work, stress by major life events, age, systolic/diastolic blood pressure, body mass index, endurance training load, depression (PHQ-9), heart-focused anxiety (HAF-17), well-being (WHO-5), physical/mental component summary in the SF-12, physical/mental health and pain in the IRES-24, self-efficacy (ASKU); at discharge from rehabiliation: Sm smoking, lifestyle change motivation, pension desire, self-assessed occupational prognosis, self-assessed health prognosis; changes during rehabilitation: systolic/diastolic blood pressure, body mass index, endurance training load, depression (PHQ-9), heart-focused anxiety (HAF-17), well-being (WHO-5), physical/mental component summary in the SF-12, physical/mental health and pain in the IRES-24, self-efficacy (ASKU).

Reviewer #1: My one main concern continues to be that since this included a strong for on an exercise-based intervention, the authors still seem to be confident on their results on changes (or in fact no change in functional capacity) purely based on the submaxmial "exercise training dose" not significantly being changed over time. This represents only a psychological desire for whatever the reason is to who and how the intensity should have been progressed over time as a very fundamental element of any exercise training programme. Unless there is a change in some measured physiological parameter like a reduction in HR for a given HR or other physiological changes demonstrating a change, the authors can not be certain that no change has occurred.

*In fact, the measured parameters of physical performance (maximum exercise capacity in the bycicle ergometry stress test, endurance training load and 6-min walking distance) showed the* ***largest*** ***enhancements*** *during cardiac rehabilitation with p-values <0.001 and standardized effect sizes up to >1 (see table 2) compared with the other measured parameters. Therefore, in our opinion, these parameters of physical performance suggested by experts as indicators of rehabilitation success were adequately confirmed by our study. Moreover, the changes in the endurance training load increase the probability of return-to-work statistically significantly, but to a small extend (OR 1.09 per 5 Watts). The other parameters of physical performance were not taken into account in the multivariable modeling due to the content overlap and less available data.*

Reviewer #2: I would like to compliment the authors on their revisions. The document has greatly improved. Nevertheless, I still have some remarks that I hope the authors are willing to take into consideration. I mainly feel that the discussion section could be further improved.

Major remarks

1. The introduction greatly improved as compared to the previous version. Nevertheless, I am still not convinced of the added value of investigating predictors of HRQL. The introduction states that there are mainly inconclusive results with regard to return to work with suboptimal outcomes after CR. Lines 85-88 seem to suggest that predictors for HRQL are already known. In line 73 the authors state that return to work influences HRQL, but why do the authors decide to separately investigate predictors of return to work AND HRQL and not just return to work?

*The relationship between return-to-work and HRQL is probably bi-directional and not causal in only one direction. There are some studies investigating predictors for occupational reintegration and HRQL, but usually not in the same study with the same set of parameters for both outcomes. Moreover, the multitude of parameters that can be modified by CR and in particular patient reported outcomes was not systematically taken into account. We added the following connecting reasoning in line 87-88:* Since return-to-work and HRQL after a cardiac event are closely associated, [12] several common predictors can be assumed. (line 87-88)

2. A clear research aim/goal is missing in the introduction

*We added the following aim:* In the OutCaRe-study we aimed to identify predictors of occupational reintegration and HRQL among the same set of patient-reported outcome measures, clinical parameters, cardiovascular risk factors and physical performance. (line 92-94)

3. In the statistics sessions the MID is mentioned for part of the patient-reported outcomes, but not for all. How did you deal with the other outcomes, such as the WHO-5, ASKU and Wurzburger list?

*The use of MID was a complementary method (in addition to statistical significance and determination of effect size) for evaluating changes in patient reported outcomes, which was added as requested by the editor. MIDs are not available for all measures. We modified the regarding sentence in the methods section as follows*: In addition, the changes in patient-reported outcome measures (metric variables) during CR were assessed using the minimal important difference (MID) if available. (line 190-191) *The MID of WHO-5 is 10% as stated below (line 193-194). Statistical significance and standardized effect size were used for the evaluation of ASKU (no MID available). The Wurzburger screening generates categorical variables, no MID exists.*

4. The discussion sessions has greatly improved with your extra information, but is now quit long. I suggest to shorten this section and also rearrange a bit the paragraphs. Start with discussing the predictors that you found and how you think (based on literature) these predictors could influence RTW/QOL (this last information is for instance missing for heart-focused anxiety). Move the paragraph lines 349-358 to the end and combine this paragraph with lines 382-389. Describe in this paragraph not only what is NOT working for RTW & HRQL, but also give clear suggestions (based on your outcomes) what should be done to improve CR.

*Thank you for your suggestion. In the first revision, we added several items to the discussion section as requested by the editor and reviewers (e.g. MID, educational level and return to work, HRQL related predictors, multicollinearity, benefit of social workers, missing data). Taking into account these several issues, we rearranged and rewrote the discussion section.*

*From our point of view, the paragraph discussing the benefit of social workers in CR is placed in a logical order to the paragraph above. Moving the paragraph would take the content out of the context.*

*In the last paragraph above the limitation section we recommend to provide multiprofessional CR programms including cardiologists, social workers and sports therapists or physiotherapists on the basis of an individualized treatment approach. For this we recommend the consistent implementation of questionnaires assessing subjective health (see conclusion). More recommendations are not supported by our data.*

5. There seems to be some grammar errors in the paper. I recommend consulting a native speaker/ professional to check the paper.

*The paper has been revised by a professional proof reader.*

Smaller remarks

*We considered all following remarks.*

1. In the abstract ‘lifestyle change motivation’ is defined as risk factor management, shouldn’t this be part of patient-reported outcomes?

2. I advise to split lines 42-46 (abstract) in two sentences to increase readability

3. Please make clear in your abstract what predictors were measured at discharge and which predictor concern changes during CR.

4. Could you add a reference to line 73 that return to work influences HRQL?

5. Line 74-77 it is unclear whether outcomes or predictors are discussed (e.g. functional status is a predictors and HRQL an outcome?). I advise to delete this sentence.

6. Please also add to your methods section that you check for multicollinearity.

7. Sex and gender are both used in your document. Please pick one (I prefer sex).

8. I would recommend adding a subheading “Predictors” to page 6 to increase readability.

9. Nice summary at the start of the discussion. Could you shortly add (one sentence) how RTW and QOL were improved at 6 months follow-up?
