# Supplementary material for: Patient-reported outcomes predict return to work and health-related quality of life six months after cardiac rehabilitation: Results from a German multi-centre registry (OutCaRe)
Source: PLoS One. 2020 May 5;15(5):e0232752. doi: 10.1371/journal.pone.0232752 (PMC7199966; doi:10.1371/journal.pone.0232752)
Supplement: Attachment — Submitted filename: 2020-04-20_OutCaRe_RTW-QoL_ReviewerResponseIII.docx [file pone.0232752.s008.docx]

**PONE-D-19-29345R2**

**Patient-reported outcomes predict return to work and health-related quality of life 6 months after cardiac rehabilitation: Results from a German multi-centre registry (OutCaRe)**

**Responses to the Editor and Reviewers’ Comments**

*We thank the editor and the reviewers for re-reviewing our manuscript. Please find our responses to the comments below. Changes in the manuscript text are highlighted in grey.*

*Sincerely,*

*Annett Salzwedel & Karl Wegscheider
for the authors of the manuscript.*

Editor:

*Thank you very much for the hints, which we’ve all considered.*

Abstract, line 27, please insert a comma before "as well as".

In the title and throughout the text, please write numbers under 10 using letters (e.g., 6 = six)

Abstract, please include '=' signs after OR and colons (:) after CI.

Abstract, line 52: please change this to "patients with heart disease".

Line 75, remove "the" at the end of this line.

Line 85, there appears to be a comma missing between exercise and coronary?

Throughout the text please change to patients living with heart disease vs. heart disease patients.

Line 91, insert a comma before "as well as".

Line 91, insert in brackets after mid-term course what you mean by this (e.g., provide time frame)

Line 92, define OutCaRe-study upon first occurrence.

Line 97, insert a comma before "as well as".

Lines 105-108: please place these items on the same line and use semicolons (;) to separate them.

Lines 129-130: please use commas instead of - to separate the training group indication. i.e. with a duration, depending on the training group and physical performance, of up to...

Line 218, define ACS

*All done.*

Lines 219-221: explain your cut-off used for VIFs

*We added the following explanation to the statistics:* For the complete set of potential regressors, potential multicollinarity was studied by calculating the variance inflation factors, values below 10 were considered acceptable. (line 217-218)

Line 235: Write out 18 (i.e. Eighteen)

Line 275: Write out 89 (i.e. Eighty nine)

Line 278-282: this sentence is too long. Consider separating into two sentences the first ending after "summary scale" on line 280 and the second beginning with "Scores for HRQL six months after CR..."

Line 281 and throughout manuscript, include = after OR

Line 292: consider revising writing to: "mental-HRQL"

Line 356, insert a comma before "as well as".

Line 433, change "whole population" to the general CR population

Line 459, insert a comma before "as well as".

Please be consistent in your writing of "return to work" vs. "return-to-work"

*All done.*

Did the authors check if their non-imputed models achieved similar results as the imputed models?

*Yes, actually we started with a non-imputed model. However, the type of model that is adequate for the problem at hand require complete data sets. Without imputation, the analysis has to be done in the markedly smaller data set of complete cases (915 (73%) of 1262 cases) which may not be representative for the whole study population. Correspondingly, the analysis of the non-imputed data resulted in another set of selected variables that makes it difficult to compare the resulting models. In such cases, the imputed model is usually thought to be more reliable than the complete case model.*
